# Two *Figla* homologues have disparate functions during sex differentiation in half-smooth tongue sole (*Cynoglossus semilaevis*)

**DOI:** 10.1038/srep28219

**Published:** 2016-06-17

**Authors:** Hailong Li, Wenteng Xu, Ning Zhang, Changwei Shao, Ying Zhu, Zhongdian Dong, Na Wang, Xiaodong Jia, Hao Xu, Songlin Chen

**Affiliations:** 1 Key Lab of Sustainable Development of Marine Fisheries, Ministry of Agriculture; Yellow Sea Fisheries Research Institute, Chinese Academy of Fishery Sciences, Qingdao 266071, China; 2 Laboratory for Marine Fisheries Science and Food Production Processes, Qingdao National Laboratory for Marine Science and Technology, Qingdao 266273, China

## Abstract

Figla is a germ-cell-specific transcription factor associated with ovary development and differentiation. In vertebrates, one transcriptional form of *Figla* is commonly found. However, besides the common form of this gene (named *Figla_tv1*), a new transcriptional form (named *Figla_tv2*) was identified in half-smooth tongue sole (*Cynoglossus semilaevis*). The full-length cDNA of *Figla_tv1* was 1057 bp long with a 591-bp open reading frame encoding a predicted 196 amino acid protein, while *Figla_tv2* encoded a 125 amino acid protein. *Figla_tv1* and *Figla_tv2* expression in various tissues was detected by qRT-PCR. *Figla_tv1* was expressed mainly in ovary, skin and liver, while *Figla_tv2* was expressed in all examined tissues. In the gonads, *Figla_tv1* was expressed in ovary, while *Figla_tv2* was predominately expressed in testis of pseudomales. Further, *in situ* hybridization located *Figla_tv1* only in oocytes and *Figla_tv2* mainly in germ cells of pseudomale testis. After knocking down *Figla_tv2* in a pseudomale testis cell line, the expression of two steroid hormone-encoding genes, *StAR* and *P450scc*, was significantly up-regulated (*P* < 0.05). Our findings suggest that *Figla_tv1* has a conserved function in folliculogenesis, as in other vertebrates, and that *Figla_tv2* may have a role in the spermatogenesis of pseudomales by regulating the synthesis and metabolism of steroid hormones.

Basic helix-loop-helix (bHLH) proteins are members of a large superfamily that regulates a number of developmental and metabolic processes, including sex determination, cell differentiation, nervous system development, oncogenesis, and cholesterol metabolism^1^,^2^. Studies in many species, including *Drosophila*, *Saccharomyces cerevisiae*, *Homo sapiens*, and *Arabidopsis*, have provided a great deal of information about the structure and function of this family of proteins^2^,^3^,^4^,^5^,^6^,^7^,^8^.

The factor in the germline alpha (*Figla*) gene encodes a germ cell-specific bHLH transcription factor that mediates several developmental processes, including fertilization and early embryogenesis^9^. Many studies have focused on the important roles of Figla in gonad development and differentiation. In *Mus musculus*, *Figla* expression was first detected in the ovary at embryo day 13 (E13)^10^. Its expression increased dramatically at the end of embryo development and peaked at two days postpartum, when oocytes have become enclosed in primordial follicles^10^, suggesting the probable involvement of Figla in ovary follicle development. Furthermore, Figla was shown to regulate the expression of three zona pellucida genes (*Zp1*, *Zp2*, and *Zp3*) that encode proteins necessary for zona pellucida formation in growing oocytes^11^, indicating a crucial role for Figla in folliculogenesis. Moreover, targeted mutations of *Figla* in female *M. musculus* resulted in abnormal ovarian gonadogenesis, including failure to form primordial follicles, massive depletion of oocytes, and subsequent female sterility^10^. However, in mutated males, gonad development appeared to be normal, and these mice were fertile^10^. Together, these data indicate that Figla is indispensable only for ovary folliculogenesis and is not essential for testis development. In a subsequent study, it was suggested that Figla may balance sexually dimorphic gene expression in the postnatal ovary because *Figla* knockout resulted in the enhanced expression of many testis-specific genes in the oocytes of newborn *M. musculus*, while in male germ cells, the ectopic expression of *Figla* down-regulated a subset of these testis-specific genes^12^.

In teleosts, *Figla* has commonly been regarded as a marker gene of ovary development or early oocyte differentiation, but studies examining the regulation and roles of *Figla* in gonad development are limited^13^,^14^,^15^. Half-smooth tongue sole (*Cynoglossus semilaevis*) is an economically important marine flatfish in China. Because of their sex-dimorphic growth (females grow 2 to 4 times faster than males^16^), increasing the proportion of females in cultivated populations will be economically beneficial. To achieve this goal, understanding the sex-determination and differentiation mechanisms of *C. semilaevis* is particularly important. The primary sex of *C. semilaevis* is determined by the sex chromosomes: females (ZW) harbor a large W sex chromosome, while males possess two ZZ sex chromosomes^17^. Approximately 14% of ZW genetic females were shown to be sex-reversed to phenotypic males, the so-called pseudomales^17^. Interestingly, these pseudomales are fertile and can mate with the normal females to produce the viable offspring. A number of sex-related genes, including *Dmrt1*, *wnt4a*, *Sox9*, *Foxl2*, *tesk1*, *piwil2*, and *Gadd45g*, have been isolated from *C. semilaevis*; however, the molecular mechanisms associated with sex determination and differentiation are still poorly understood^18^,^19^,^20^,^21^,^22^,^23^. In a previous study, we identified two different transcripts of *Figla* (hereby named *Figla*, *transcript variant 1* [*Figla_tv1*] and *Figla*, *transcript variant 2* [*Figla_tv2*]) in our RNA-seq data from adult gonads of *C. semilaevis* and found that the methylation levels of these genes were closely related to gonad development^24^. However, the gonad expression patterns and functions of the two *Figla* transcripts during development are still unclear. In the present study, we cloned the full-length cDNAs of the two *Figla* isoforms in *C. semilaevis* by rapid amplification of cDNA ends (RACE) and used quantitative real-time polymerase chain reaction (qRT-PCR) and *in situ* hybridization (ISH) to detect the spatial and gonad expression of the two genes. Furthermore, the expression of genes that may be regulated by *Figla_tv2* was analyzed after RNA interference (RNAi) knockdown of *Figla_tv2*. In the present study, we aimed to identify the sequences of the *Figla_tv1* and *Figla_tv2* transcripts, examine their sex-dimorphic expression profiles, and illustrate their functional diversity during gonad development.

## Results

### Sequence characteristics of two *Figla* homologues in *C. semilaevis*

In order to characterise the *Figla* homologues in *C. semilaevis*, we successfully isolated 5′- and 3′-RACE fragments of *Figla_tv1* and *Figla_tv2* from adult ovary and pseudomale testis of *C. semilaevis* and assembled two full-length cDNAs. The cDNA sequences have been deposited in GenBank with accession numbers KT966740 (*Figla_tv1*) and KT966741 (*Figla_tv2*).

The full-length cDNA of *Figla_tv1* was 1050 bp long with an open reading frame of 591 bp encoding a 196 amino acid (aa) protein, and the 3′ and 5′ untranslated regions (UTRs) were 317 bp and 142 bp, respectively (Figure S1A). The putative Figla_tv1 protein was 22.2 kDa with a theoretical isoelectric point (pI) of 4.76. The full-length cDNA of *Figla_tv2* was 1510 bp long with an open reading frame of 378 bp encoding a 125 aa protein, and the 3′ and 5′ UTRs were 317 bp and 815 bp, respectively. A single typical polyadenylation signal (AATAAA) site was found in the 3′ UTR of *Figla_tv2* between nucleotides 1460 and 1465, 19 bp upstream of the poly (A) tail (Figure S1B). The putative Figla_tv2 protein was 14.0 kDa with a theoretical pI of 4.24.

**Figure 1:**
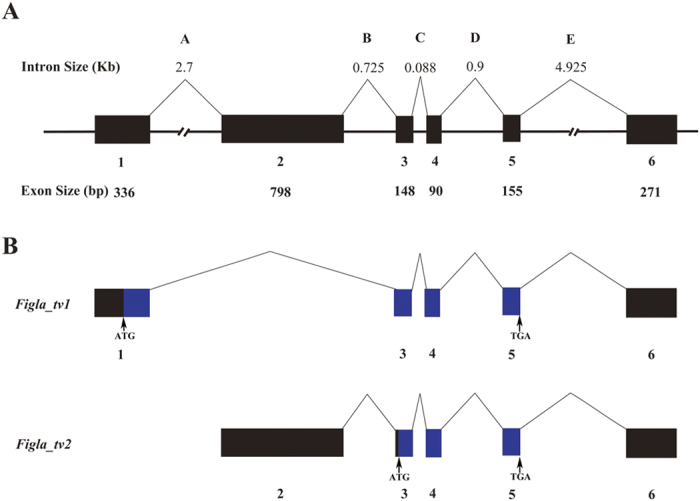
Schematic representation of the *C. semilaevis Figla* genomic structure and splice variants. (**A**) The genomic structure of the *C. semilaevis Figla* gene. The six exons numbered under the solid boxes represent the *Figla* transcribed regions, and introns indicated by letters above the lines show the alternative splicing. (**B**) The alternatively spliced *Figla* mRNA isoforms. The transcribed nucleotides are shown with boxes, while the coding nucleotides are indicated with blue boxes. The locations of the initiation codons (ATG) and stop codons (TGA) are shown by vertical arrows.

To analyse the similarities/differences between the two *Figla* nucleotide sequences, a multiple sequence alignment was conducted by Clustal X. The 714-bp nucleotide sequences at the 3′-ends were nearly identical in the two transcripts, while the remaining sequences did not show much similarity (Figure S2). In order to illustrate the alternative splicing of the two transcripts, the cDNA sequences were successfully mapped to the genomic sequence. As shown in Fig. 1A, the genomic sequence of *Figla* was comprised of six exons (1–6) that were separated by five introns (A–E). The two transcripts were produced by alternatively splicing the different exons. *Figla_tv1* was generated by splicing out the second exon, while *Figla_tv2* was generated by splicing out the first exon, and the other exons were spliced together (Fig. 1B).

**Figure 2:**
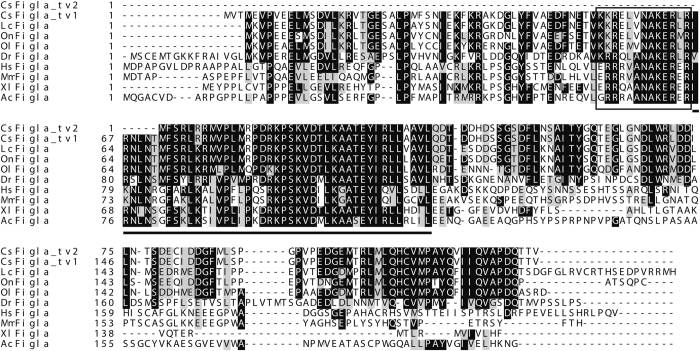
Multiple sequence alignment of Figla protein sequences from *C. semilaevis* and other vertebrates. Sequences were aligned using Clustal X (Version 2.0) and the identical or similar amino acids are shaded by BOXSHADE. The presumed basic region is boxed, and the helix-loop-helix (HLH) region is underlined. Gaps introduced in the sequences to optimize the alignment are indicated by dashes. The abbreviated species names and their GenBank accession numbers are as follows: Cs Figla_tv2: *Cynoglossus semilaevis* Figla_tv2 (KT966741); Cs Figla_tv1: *Cynoglossus semilaevis* Figla_tv1 (KT966740); Lc Figla: *Larimichthys crocea* Figla (KKF33048.1); On Figla: *Oreochromis niloticus* Figla (NP_001298259.1); Dr Figla: *Danio rerio* Figla (NP_944601.2); Ol Figla: *Oryzias latipes* Figla (NP_001098215.1); Hs Figla: *Homo sapiens* Figla (NP_001004311.2); Mm Figla: *Mus musculus* Figla (NP_036143.1); Xl Figla: *Xenopus laevis* Figla (NP_001088667.1); Ac Figla: *Anolis carolinensis* Figla (XP_008120436.1).

To evaluate the similarities/differences of vertebrate Figla proteins, a multiple sequence alignment including Figlas from *C. semilaevis* and other vertebrates was conducted. All the vertebrate Figla proteins, including Figla_tv1 from *C. semilaevis*, had a typical conserved bHLH region composed of 57 aa (Fig. 2). However, Figla_tv2 from *C. semilaevis* lacked the basic region and six N-terminal amino acids from the HLH region (Fig. 2). The two translated Figla amino acid sequences from *C. semilaevis* shared high identities with proteins from other species as analyzed by MegAlign. The bHLH region from Figla_tv1 had especially high similarity to other amino acid sequences and shared 84.2%, 96.5%, 94.7%, 93.0%, 66.7%, 66.7%, 56.1%, and 56.1% identities with sequences from *Acanthopagrus schlegelii*, *Dicentrarchus labrax*, *Oreochromis niloticus*, *Epinephelus coioides*, *Xenopus laevis*, *Chelonia mydas*, *Rattus norvegicus*, and *H. sapiens*, respectively. Figla_tv2 shared 77.6%, 77.6%, 76.8%, 71.2%, 27.2%, 22.4%, 18.4% and 18.4% identities with *Larimichthys crocea*, *O. niloticus*, *Pagrus major*, *D. labrax*, *X. laevis*, *C. mydas*, *R. norvegicus*, and *H. sapiens*, respectively.

### Tissue expression of *Figla_tv1* and *Figla_tv2*

**Figure 3:**
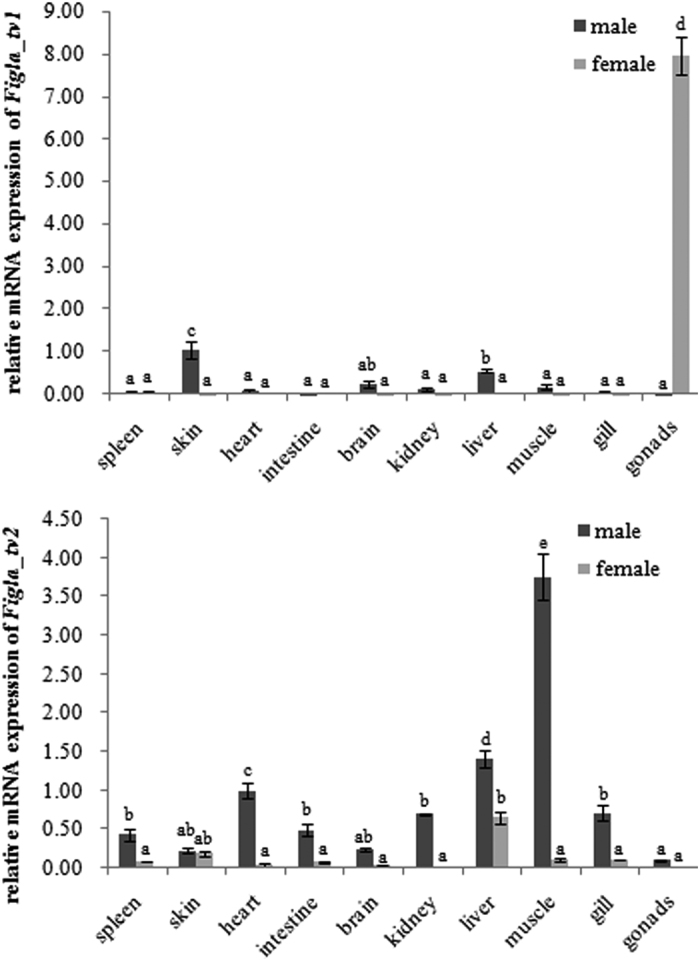
Expression levels of *Figla_tv1* and *Figla_tv2* mRNAs in *C. semilaevis* tissues evaluated by qRT-PCR. The expression levels were normalized using the geometric mean of the levels of two internal control genes (*β-actin* and *Rpl13α*). The mean ± SEM values from three separate individuals (n = 3) are shown. Bars with different letters indicate statistically significant differences (*P* < 0.05).

To determine the expression patterns of the two *Figla* isoforms, we measured mRNA levels in 11 tissues of *C. semilaevis. Figla_tv1* mRNA was predominately expressed in the skin, liver and brain of male and the gonad of female *C. semilaevis* and was negligibly expressed in the other tissues examined (Fig. 3). On the other hand, *Figla_tv2* mRNA was expressed widely in the tissues of both sexes, including skin, liver, muscle and gill (Fig. 3), although there were some differences between the tissues from males and females.

### Sexual-dimorphic expression of *Figla_tv1* and *Figla_tv2* during gonad development

**Figure 4:**
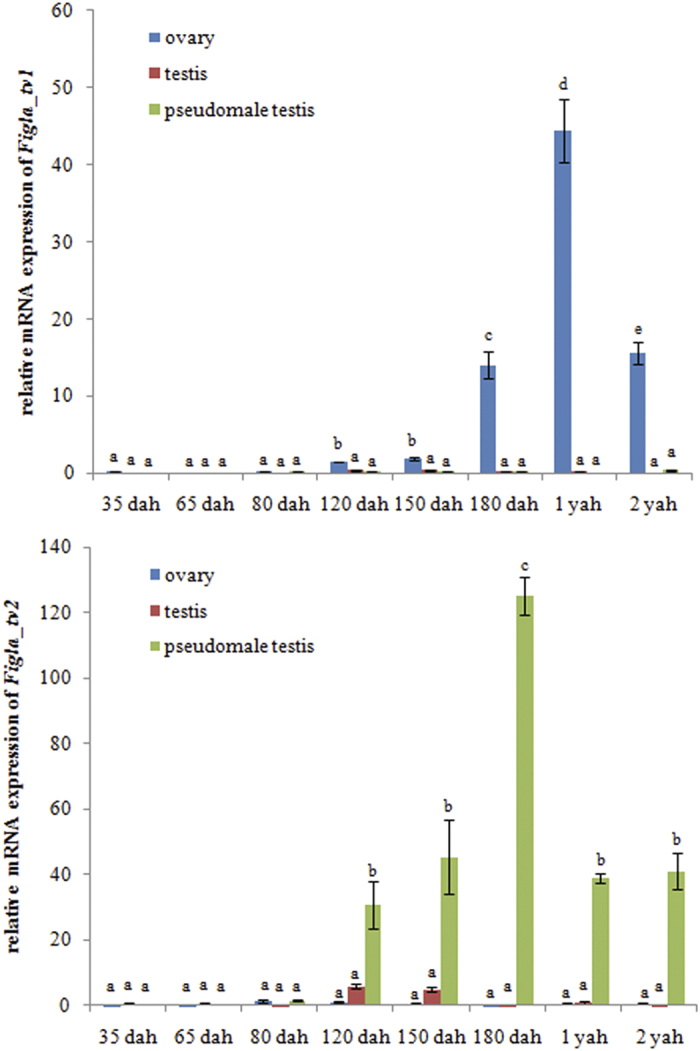
Relative expression levels of *Figla_tv1* and *Figla_tv2* at different development stages in gonads of *C. semilaevis*. The expression level was normalized using the geometric mean of the levels of two internal control genes (*β-actin* and *Rpl13α*). The mean ± SEM values from three separate individuals (n = 3) are shown. Bars with different letters indicate statistically significant differences (*P* < 0.05).

To measure the expression levels during the developmental stages of gonad differentiation, *Figla_tv1* and *Figla_tv2* expression was detected in ovary, testis and pseudomale testis at different stages by qRT-PCR. As shown in Fig. 4, *Figla_tv1* mRNA was first detected at 120 days after hatching (dah) in the ovary and then continued to be expressed until it reached a maximum at 1 year after hatching (yah) before declining sharply at 2 yah. Conversely, *Figla_tv1* mRNA was almost undetectable in the gonads of both male and pseudomale *C. semilaevis* during all the developmental stages tested (Fig. 4). *Figla_tv2* mRNA was exclusively expressed in the pseudomale testis (Fig. 4), where it was initially detected at 120 dah and then rapidly increased to reach a maximum at 180 dah before declining sharply at 1 yah. *Figla_tv2* mRNA persisted in the adult testis at 2 yah (Fig. 4).

### Cyto-location of *Figla_tv1* and *Figla_tv2* in the gonads of *C. semilaevis*

**Figure 5:**
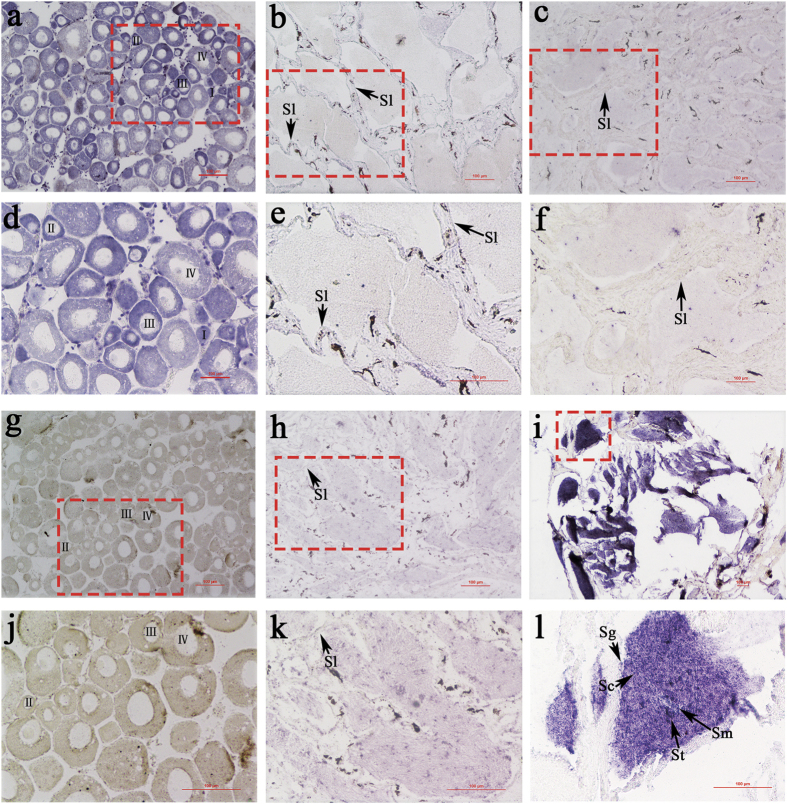
Cyto-locations of *Figla_tv1* and *Figla_tv2* mRNAs in 1 yah gonads of *C. semilaevis*. The figure shows the ovaries (left hand column), testes (middle column) and pseudomale testes (right hand column), labelled with *Figla_tv1* antisense (**a–f**) and *Figla_tv2* antisense (**g–l**) probes. (a–c) and (g–i) show the architecture with low magnification, while (**d–f**) and (**j–l**) indicate the red framed areas in (**a–c,g–i**) with large magnification. Oocytes at different developmental stages are marked by I, II, III and IV. Sg: spermatogonia; Sc: spermatocyte; St: spermatid; Sm: sperm; Sl: seminal lobule. Scale bars: 100 μm.

The cyto-location of *Figla_tv1* and *Figla_tv2* was detected by *in situ* hybridization (ISH). Ovaries from one-year-old fish contain somatic cells and oocytes at different developmental stages (stages I–IV). The ISH results showed that *Figla_tv1* was expressed in the oocytes at all four developmental stages. Strong hybridization signals were detected in the oocytes at stages I, II and III, and only faint signals were detected in the oocytes at stage IV (Fig. 5a,d) compared with the controls (Figure S3a,d). In contrast to the intense signals in the ovary, no specific signals were detected in the testis of both males (Fig. 5b,e) and pseudomales (Fig. 5c,f) compared with controls (Figure S3b,e; Figure S3c,f), which was consistent with the weak or no *Figla_tv1* expression in males and pseudomales at the 1 yah stage (Fig. 4). Testes from males and pseudomales contain germ cells from different developmental stages, including spermatogonia, spermatocytes, spermatids, and sperm. ISH revealed that *Figla_tv2* mRNA was expressed mainly in the germ cells of pseudomale testis, with strong signals detected in spermatogonia, spermatocytes, spermatids, and sperm (Fig. 5i,l) compared with the controls (Figure S3i,l). No obvious hybridization signals were observed in ovary (Fig. 5g,j) and testis (Fig. 5h,k) compared with the controls (Figure S3g,j; Figure S3h,k).

### RNAi-mediated *Figla_tv2* knockdown in *C. semilaevis*

**Figure 6:**
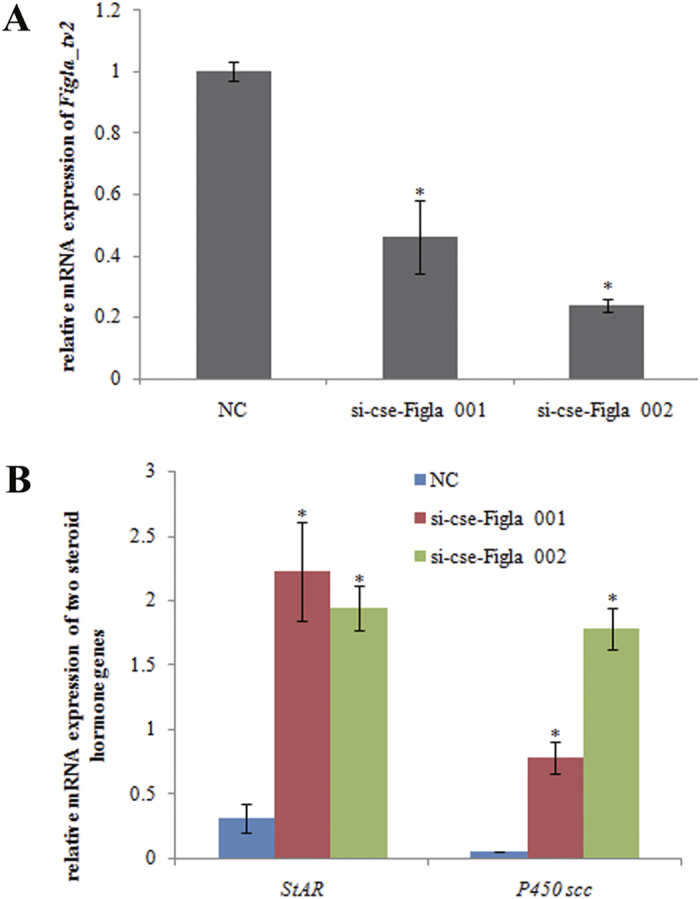
Relative mRNA expression levels of *Figla_tv2*, *StAR* and *P450scc* in cultured pseudomale gonad cells after RNAi treatment. (**A**) Expression of *Figla_tv2* after the transfection of the siRNAs for 48 h. (**B**) Expression of *StAR* and *P450scc* after the transfection of the siRNAs for 48 h. The transcripts of the *β-actin* and *Rpl13α* genes were used as internal controls to normalize the expression. NC, si-cse-Figla 001 and si-cse-Figla 002 indicate the gonad cells transfected with the siRNAs of the negative control (NC), si-cse-Figla 001 and si-cse-Figla 002, respectively. Asterisks above the bars indicate significant differences (*P* < 0.05) between the treated group and the control.

Due to no suitable cell line for RNAi of *Figla_tv1* established in *C. semilaevis*, we are unable to knock down the *Figla_tv1* gene *in vitro*. The RNAi experiment was only conducted for *Figla_tv2* gene in a *C. semilaevis* pseudomale gonad (CSPMG) cell line. To evaluate the silencing effects of RNAi, *Figla_tv2* gene expression was detected at 48 h after siRNA transfection and was found to be reduced by approximately 54% in the si-cse-Figla 001-treated group (*P* < 0.05) and 76% in the si-cse-Figla 002-treated group (*P* < 0.05) compared with the control (Fig. 6A).

### Effects of *Figla_tv2* gene silencing on the expression of two steroid hormone encoding genes

To determine the effects of *Figla_tv2* gene silencing on the expression of other sex-related genes, we measured the expression of *Sox9a*, *wt1a* and *tesk1*, three spermatogenesis-related genes in *C. semilaevis*^20^,^21^,^25^. However, no differential expression (*P* > 0.05) between the treated group and the control was observed for the three genes as detected by qRT-PCR (data not shown). Besides, the mRNA levels of two steroid hormone-encoding genes, steroidogenic acute regulatory protein (*StAR*) and cytochrome P450 side-chain cleavage (*P450scc*), were detected after RNAi silencing of *Figla_tv2* expression. As shown in Fig. 6B, the expression levels of *StAR* and *P450scc* were strongly altered. *StAR* expression increased by approximately 6-fold in the si-cse-Figla 001-treated group and by 5-fold in the si-cse-Figla 002-treated group compared with the control (*P* < 0.05), and *P450scc* expression increased by approximately 15- and 35-fold (*P* < 0.05) in the si-cse-Figla 001- and the si-cse-Figla 002-treated groups, respectively, compared with the control (Fig. 6B).

## Discussion

The *Figla* gene has been cloned in many mammalian and fish species, but until now only one transcript has been reported in each species. In the present study, in addition to the common form (*Figla_tv1*) of the gene, we isolated and characterized another transcript (*Figla_tv2*) from *C. semilaevis*. The highly conserved bHLH region is thought to be required for Figla binding to the E-box motif of the target protein^10^,^11^. Because Figla_tv2 lacked the conserved bHLH region (Fig. 2), we speculate that Figla_tv2 may be incapable of interacting with DNA directly and may function by associating with other molecules.

In vertebrates such as *Bos taurus*, *M. musculus*, *O. latipes*, *O. niloticus* and *H. sapiens*, *Figla* was expressed mainly in the gonads^9^,^11^,^13^,^15^,^26^. In this study, we also detected *C. semilaevis Figla_tv1* expression mainly in the gonads of one-year-old female fish (Fig. 3), indicating the conserved role of Figla_tv1 in gonad tissue during development of the fish. We also noted that *Figla_tv1* was expressed in other tissues, such as the skin and liver of male *C. semilaevis* (Fig. 3). However, the function of *Figla* in these tissues is not yet clearly understood. The robust expression of *Figla* in the skin and liver of *C. semilaevis* may indicate a role for Figla in immune functions, as previously reported in several teleosts^27^,^28^,^29^. It is very likely that the previous studies of *Figla* tissue distribution reported overlapping expression of both alternative splicing forms. For example, *G. rarus Figla* showed a similar composition to *C. semilaevis Figla_tv1*, but its expression pattern was the same as *Figla_tv2* (Fig. 3)^14^. To evaluate whether there are multiple *Figla* isoforms in other teleosts too, we searched the transcriptome data in GenBank and Ensemble. We found that in most teleosts there was only one *Figla* isoform (*Figla_tv1*) except that *Takifugu rubripes* expressed three *Figla* isoforms. Therefore, we speculate the multiple *Figla* isoforms probably exist in some fish species or even are generally present in the teleosts, while they are undetectable due to the sequencing depth or coverage. More studies are needed to test these possibilities.

In *C. semilaevis*, the histological and cellular differentiation of the ovary is not synchronous; histological differentiation was reported to begin 56–62 dah, whereas cellular differentiation occurred at 120 dah with the appearance of the ovarian cavity^18^. In this study, *Figla_tv1* mRNA was first detected at 120 dah in the ovary and persisted into adulthood (Fig. 4). This finding is reasonably consistent with the cellular differentiation period of the ovary, suggesting that *Figla_tv1* may play a crucial role in ovary differentiation. In addition, qRT-PCR and ISH both detected dimorphic expression of *Figla_tv1* in the gonads, with strong expression in the ovary during development and negligible expression in the testis (Figs 4 and 5). This result is consistent with previous reports in *M. musculus*, *O. latipes*, *O. niloticus*, and *H. sapiens*^11^,^13^,^15^,^26^. However, the ectopic expression of *Figla* in *O. niloticus* XY fish resulted in the depletion of germ cells and down-regulation of spermatogenesis-associated genes, suggesting Figla may play an important role in ovarian development by repressing the expression of spermatogenesis-associated genes^15^. Based on similar results with *Figla_tv1* in *C. semilaevis*, we speculate that Figla_tv1 may be involved in ovary folliculogenesis.

In order to evaluate whether *Figla_tv2* was involved in sex-reversal, we measured the levels of *Figla_tv2* transcripts in the gonads of males, females and pseudomales. *Figla_tv2* was expressed exclusively in the testis of pseudomales, with weak or no expression in the testis of males or in the ovary of females (Fig. 4), suggesting it is probably a pseudomale-biased gene that may play an important role in the sex-reversal process.

The ISH results showed that *Figla_tv2* mRNA was mainly located in the germ cells of pseudomale testis (Fig. 5i,l), suggesting an important role in regulating spermatogenesis. However, the underlying regulatory mechanism is still unknown. Proteins that belong to the bHLH superfamily generally function as transcriptional enhancers or inhibitors of target genes^1^,^2^. Despite the lack of a complete bHLH region, we speculate that Figla_tv2 may regulate the development of pseudomale testis by repressing or activating its target genes. Steroid hormones are commonly present in the gonads of vertebrates, where they are known to play critical roles in gonad development^30^,^31^,^32^. StAR and P450scc are key regulatory proteins in the synthesis and metabolism of steroid hormones in teleosts^33^,^34^,^35^. StAR mediates the transport of cholesterol across the mitochondrial membrane, a rate-limiting step in steroid hormone synthesis^32^,^33^,^34^,^35^, and P450scc catalyzes the conversion of cholesterol to pregnenolone, a pivotal step in the initiation of steroidogenesis^33^,^34^,^35^. After the *in vitro* knockdown of *Figla_tv2* in cultured pseudomale testis cells, qRT-PCR showed that *StAR* and *P450scc* mRNA levels were significantly up-regulated (Fig. 6B), suggesting *C. semilaevis Figla_tv2* may be involved in spermatogenesis by regulating steroid hormones synthesis and metabolism in the gonads of pseudomales.

## Conclusions

Two functional homologues, *Figla_tv1* and *Figla_tv2*, were successfully isolated from *C. semilaevis*. The two transcripts had different tissue expression patterns in males and females, implying their probably functional diversity. *Figla_tv1* exhibited predominant “vertebrate-like” ovarian expression, suggesting a conserved role in folliculogenesis. *Figla_tv2* was expressed mainly in the testis of pseudomales and is likely to play a role in the spermatogenesis of pseudomales by influencing the synthesis and/or metabolism of steroid hormones.

## Methods

### Ethical statement

The collection and handling of the animals used in this study was approved by the Animal Care and Use Committee at the Chinese Academy of Fishery Sciences, and all the experimental procedures were performed in accordance with the guidelines for the Care and Use of Laboratory Animals at the Chinese Academy of Fishery Sciences.

### Fish and sample collection

All half-smooth tongue sole (*C. semilaevis*) used in these experiments were purchased from Huanghai Aquaculture Ltd. (Haiyang, China). One-year-old fish (three individuals of each sex) were randomly sampled. Spleen, skin, heart, intestine, brain, head kidney, liver, muscle, gill, ovary, and testis tissues (11 in all) were collected and immediately transferred to liquid nitrogen and were then stored at −80 °C until RNA extraction. In addition, parts of the gonad were fixed in 4% paraformaldehyde (pH 7.4) at 4 °C for 24 h, then dehydrated through a methanol series (25, 50, 75, and 100%) and finally stored in 100% methanol at −20 °C for ISH analysis. Moreover, three independent gonadal samples at 35, 65, 80, 120, 150, and 180 dah and at 1 and 2 yah were sampled within one family. To determine the genetic sex of the fish, parts of the caudal fins were collected and fixed in 100% ethanol for DNA extraction.

### cDNA synthesis and identification of the genetic sex

Total RNA was extracted from the frozen tissues with TRIzol reagents (Invitrogen, Carlsbad, CA, USA) according to the manufacturer’s instructions. Then, the RNA was transcribed to cDNA using a PrimeScript™ RT reagent kit (TaKaRa Bio Inc., Otsu, Japan) with gDNA Eraser to avoid contamination by genomic DNA.

Genomic DNA was extracted from the fins using the phenol-chloroform method as described previously^16^. Female-specific primer pairs (CseF382F and CseF382R) (Table S1) were used to identify the genetic sex of the fish^23^. After PCR amplification with the conditions: 94 °C for 5 min, followed by 35 cycles (94 °C for 30 s, 54 °C for 30 s, and 72 °C for 30 s), and 72 °C for 10 min, the products were run on a 1.2% agarose gel, and samples with a single 291 bp band were identified as genomic females, while samples with no bands were identified as genomic males.

Phenotypic sex was determined by histological analysis following procedures described previously^36^. Pseudomales are individuals for which the genomic sex is identified as female, but the phenotypic sex is male.

### Isolation of *Figla* full-length cDNA from *C. semilaevis* tissues

RACE-ready first-strand cDNA was synthesized from total RNA using a SMART™ RACE cDNA amplification kit (Invitrogen) according to the manufacturer’s instructions. The specific primers for the outer and nest amplifications were based on the mRNA sequence that determined from whole-genome sequencing^18^ and the RNA-seq data^24^ of *C. semilaevis*. The sequences of all primers used for RACE amplifications are listed in Table S1. The universal primer UPM and the outer primers were used for the first 5′- and 3′-RACE amplifications. The products were then diluted 100 times with ddH_2_O and used as templates for the nested PCR reactions with the nest primers and the universal primer NUP. The outer and nest amplifications were both performed using the same touchdown PCR procedures: denaturation at 94 °C for 5 min, followed by 15 cycles (94 °C for 30 s, first five cycles 68 °C for 30 s, and subsequently the temperature was reduced by −2 °C per five cycles, and 72 °C for 2 min), 20 cycles (94 °C for 30 s, 60 °C for 30 s, and 72 °C for 2 min), and then incubation at 72 °C for 10 min. The nested PCR products were separated on a 1.2% agarose gel and purified with a DNA purification kit (TaKaRa Bio Inc.). The purified 5′- and 3′-RACE products were subcloned into a PMD-18T vector (TaKaRa Bio Inc.) and sequenced.

### Bioinformatics analysis

The 5′- and 3′-RACE fragments were assembled using DNASTAR software (DNASTAR, Madison, WI) to obtain the full-length cDNA sequences of *Figla_tv1* and *Figla_tv2.* Sequence identity and similarity of the two translated Figla proteins from *C. semilaevis* with known Figla proteins from other vertebrates were assessed using the MegAlign program (DNASTAR) with Clustal W method. Sequence alignments of two *Figla* nucleotide sequences were generated using the Clustal X^37^ and GeneDoc programs^38^. Multiple sequence alignments of Figla proteins from different species were conducted using the Clustal X^37^ and edited by BOXSHADE (http://www. ch.embnet.org/software/BOX_form.html).

### qRT-PCR analysis

The expression levels of the two *Figla* genes in the tissues and gonads at differential developmental stages were determined by qRT-PCR as described previously^23^. Two pairs of primers (Table S1) were designed based on the specific gene regions of *Figla_tv1* and *Figla_tv2*. The specificity of the primers was verified by a single distinct peak obtained in a melting curve analysis. The *β-actin* was identified previously as a reliable internal reference gene in various tissue samples of *C. semilaevis*^39^ and was used as the first reference gene. In addition, *Rpl13α* was validated as a suitable reference gene for quantifying gene expression in teleost species such as *Danio rerio*^40^. Our recent studies have found it was expressed with a relatively stable level in various tissues of *C. semilaevis* (data not shown). Therefore, it was employed as the second reference gene. The geometric means of *β-actin* and *Rpl13α* expression levels were used to normalize the data. Quantification was conducted using an ABI 7500 detection system (Applied Biosystems, Foster City, CA, USA) with SYBR Green Master Mix (TaKaRa Bio Inc.). The qRT-PCR procedure was as follows: 94 °C for 30 s, followed by 40 cycles of 94 °C for 5 s, and 60 °C for 34 s. Tissues and gonads were sampled from three individuals, and triplicate assays for each sample were conducted. The ABI 7500 system SDS software version 1.4 (Applied Biosystems) was used to analyze the qRT-PCR data, and baseline and cycle threshold values were set automatically. The relative mRNA expression of target genes was calculated by the 2^−△△Ct^ method, as described previously^41^.

All data are presented as the mean ± SEM of three samples with three parallel repetitions. Differences between means were tested by one-way analysis of variance (ANOVA) followed by Duncan’s post-hoc test. (SPSS software version 18.0; SPSS Inc., Chicago, IL), and the significance level was set at *P* < 0.05. All assays in the qRT-PCR complied with the MIQE guidelines^42^.

### *In situ* hybridization

To synthesize digoxigenin (DIG)-labeled RNA sense and antisense probes, the cDNA fragments of *Figla_tv1* and *Figla_tv2* were amplified by PCR using primer pairs Figla_tv1-ISH-F (*Bam*HI site underlined) and Figla_tv1-ISH-R (*Eco*RV site underlined) and Figla_tv2-ISH-F (*Bam*HI site underlined) and Figla_tv2-ISH-R (*Eco*RV site underlined) (Table S1), respectively. The PCR products were sub-cloned into the pEASY-T5-Zero vector and verified by sequencing. The recombinant plasmids with the correct insertion were extracted with a plasmid extraction kit (Tiangen Bio Inc., Beijing, China) and linearized by *Bam*HI or *Eco*RV. The linearized plasmids were used as templates and the probes were synthesized with a DIG RNA Labeling Kit (Roche, Mannheim, Germany). For ISH analysis, the stored gonads were dehydrated in an ascending gradient of ethanol and embedded in paraffin wax, and 6-μm sections were cut. ISH was performed as described previously^43^ using three replicate samples. The sections were examined and photographed with a Nikon E80i microscope (Nikon Co., Tokyo, Japan).

### RNAi silencing of *Figla_tv2* and *in vitro StAR* and *P450scc* expression

Two *Figla_tv2*-specific small interfering RNAs (siRNAs), si-cse-Figla 001 and si-cse-Figla 002, and a nonspecific siRNA negative control (NC) were designed and synthesized by RayBiotech Co., Ltd. (Guangzhou, China). The cells used for the RNAi treatment were from the CSPMG cell line that was established previously^44^ and stored in liquid nitrogen. After thawing by incubating in a 42 °C water bath for 5 min, the CSPMG cells were harvested by centrifugation at 180 × g for 10 min. Then, the cells were incubated at 24 °C in 25-cm^2^ cell culture flasks with 20% FBS-DMEM/F12 medium. After an approximately 3 days of culturing, the cells formed a confluent monolayer. The cells were transferred to six-well plates and cultivated at 24 °C for 12 h to ensure complete attachment to the plates. The siRNA was transfected into the CSPMG cells using Lipofectamine 2000 reagent (Invitrogen) following the manufacturer’s instructions. An NC siRNA labeled by Cy3 was used to assess the transfection efficiency, and the transfection efficiency was evaluated by calculating the ratio of cells expressing red fluorescence signals to all the cells used for transfection. The average transfection efficiency of the cells used for assay was calculated to be about 90%. The treated groups were transfected with siRNA (si-cse-Figla 001 and si-cse-Figla 002) at a final concentration of 30 nM. The controls were transfected with NC siRNA at the same concentration. The cells from the treated and control groups were cultivated at 24 °C for 48 h and were then harvested by centrifugation at 180 × g for 10 min. After washing three times with phosphate-buffered saline, the cells were stored in the same TRIzol reagents that were used for the RNA extraction. Three replicates were conducted for each group. Total RNA extraction and the re-transcriptions were performed according to the methods described above. The expression of *Figla_tv2*, *StAR*, and *P450scc* was measured by qRT-PCR using the primers listed in Table S1. The qRT-PCR data were analyzed as described above.

## Additional Information

**How to cite this article**: Li, H. *et al.* Two *Figla* homologues have disparate functions during sex differentiation in half-smooth tongue sole (*Cynoglossus semilaevis*). *Sci. Rep.* **6**, 28219; doi: 10.1038/srep28219 (2016).

## Supplementary Material

Supplementary Information
