# Supplementary material for: Two Figla homologues have disparate functions during sex differentiation in half-smooth tongue sole (Cynoglossus semilaevis)
Source: Sci Rep. 2016 Jun 17;6:28219. doi: 10.1038/srep28219 (PMC4911598; doi:10.1038/srep28219)
Supplement: Supplementary Information [file srep28219-s1.pdf]

Supplementary information

Two *Figla* homologues have disparate functions during sex differentiation in half-smooth tongue sole (*Cynoglossus semilaevis*)

Hailong Li[1, 2], Wenteng Xu[1, 2], Ning Zhang[1, 2], Changwei Shao[1, 2], Ying Zhu[1, 2], Zhongdian Dong[1, 2], Na Wang[1, 2], Xiaodong Jia[1, 2], Hao Xu[1, 2] & Songlin Chen[1, 2]*

[1] Key Lab of Sustainable Development of Marine Fisheries, Ministry of Agriculture; Yellow Sea Fisheries Research Institute, Chinese Academy of Fishery Sciences, Qingdao 266071, China

[2] Laboratory for Marine Fisheries Science and Food Production Processes, Qingdao National Laboratory for Marine Science and Technology, Qingdao 266273, China

***Corresponding author:

E-mail: chensl@ysfri.ac.cn

Supplementary Figures

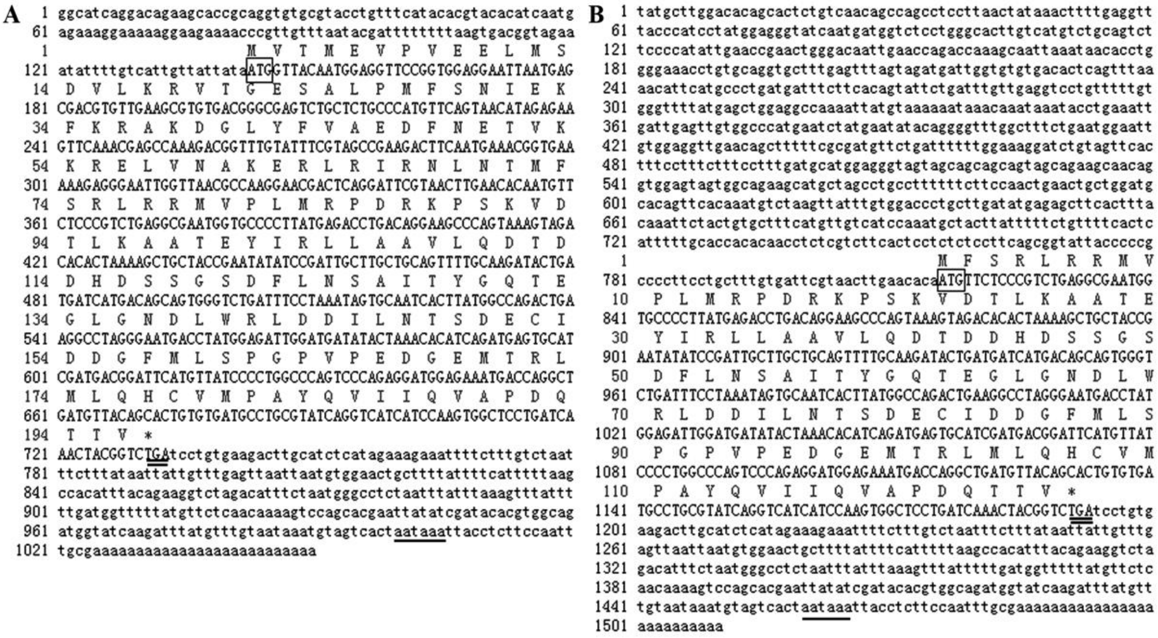

Figure S1 Full-length cDNA sequences and deduced amino acid sequences of *Figla_tv1* (A) and *Figla_tv2* (B) from *C. semilaevis*. Lower case letters indicate the 5′ and 3′ UTR sequences. Upper case letters indicate the coding sequence. The predicted start codon (ATG) is boxed, and the predicted stop codon (TGA) is double underlined. The polyadenylation signal (aataaa) is underlined.

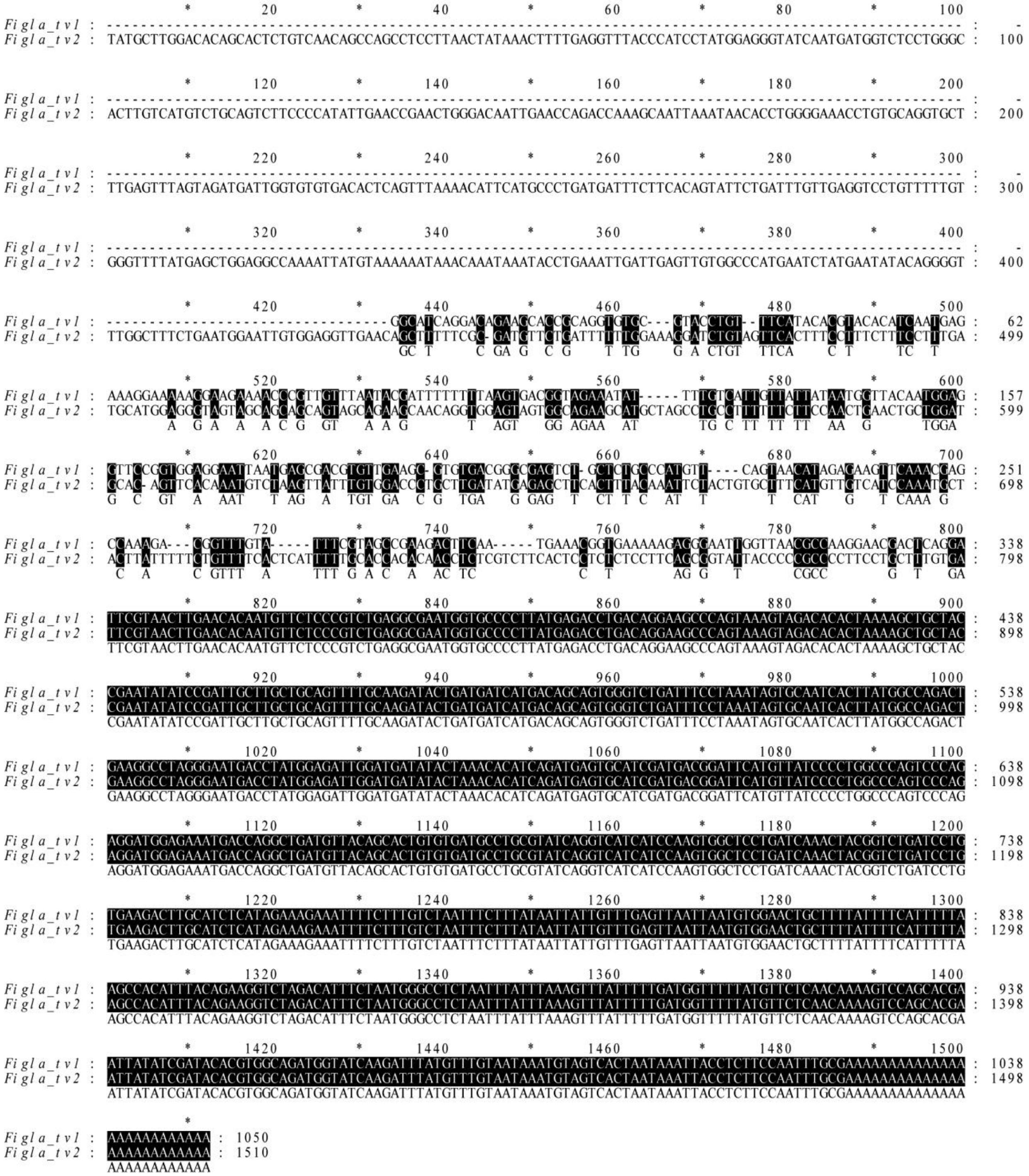

Figure S2 Nucleotide sequence alignment of two *Figla* cDNAs from *C. semilaevis*.

Sequences were aligned by Clustal X and edited by GeneDoc. Identical amino acids are indicated by black shading. Gaps introduced in the sequences to optimize the alignment are indicated by dashes.

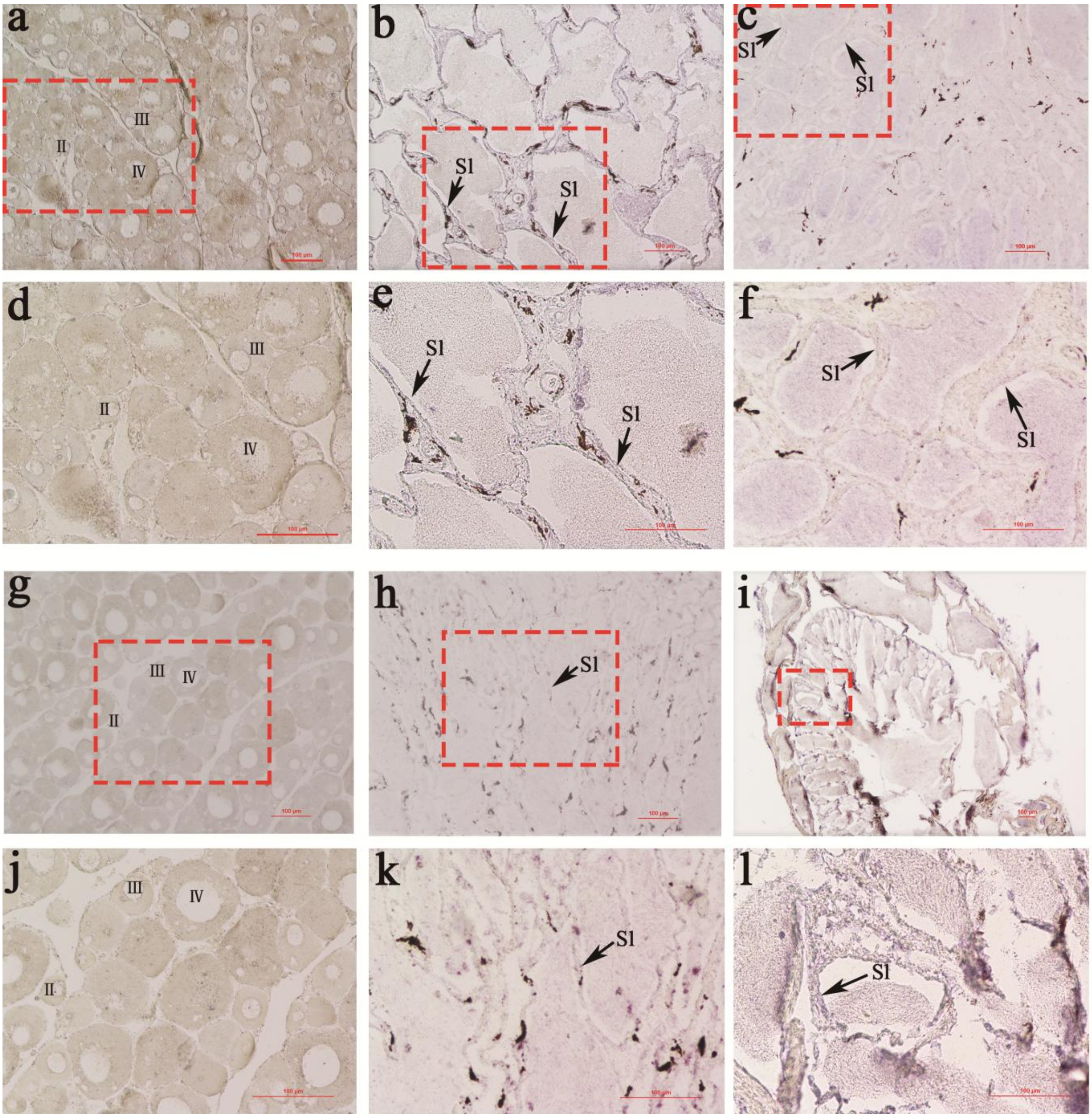

Figure S3 Cyto-locations of *Figla_tv1* and *Figla_tv2* mRNAs in 1 yah gonads with sense RNA probes. The figure shows the ovaries (left hand column), testes (middle column) and pseudomale testes (right hand column), labelled with *Figla_tv1* sense (a-f) and *Figla_tv2* sense (g-l) probes. a-c and g-i show the architecture with low magnification, while d-f and j-l indicate the red framed areas in (a-c, g-i) with large magnification. Oocytes at different developmental stages are marked by II, III and IV.

Sl: seminal lobule. Scale bars: 100 μm.

Supplementary Tables

Table S1 Primers used in the PCR amplifications.

Group	Primers name	Sequence (5'-3')
Sex identification primers	CseF382F	ATTCACTGACCCCTGAGAGC
	CseF382R	TGGCACCATCATTGTAAAACTA
RACE primers	Figla_tv1-5'R	CTGGTCATTTCTCCATCCTCTGGGACTGG
	Figla_tv1-5'N	GGGGCACCATTCGCCTCAGACGGGAGAAC
	Figla_tv1-3'R	TGTTCTCCCGTCTGAGGCGAATGGTGCC
	Figla_tv1-3'N	CCCAGTCCCAGAGGATGGAGAAATGACC
	Figla_tv2-5'R	AGTTACGAATCACAAAGCAGGAAGGGGC
	Figla_tv2-5'N	CGCTGAAGGAGAGAGGAGTGAAGACGAG
	Figla_tv2-3'R	TATGGCCAGACTGAAGGCCTAGGGAAT
	Figla_tv2-3'N	GGCTGATGTTACAGCACTGTGTGATGC
	UPM-long	CTAATACGACTCACTATAGGGCAAGCAG<br>TGGTATCAACGCAGAGT
	UPM-short	CTAATACGACTCACTATAGGG
	NUP	AAGCAGTGGTATCAACGCAGAGT
qRT-PCR primers	Figla_tv1-qRT-F	ACATAGAGAAGTTCAAACGAGCC
	Figla_tv1-qRT-R	CGGTAGCAGCTTTTAGTGTGTCT
	Figla_tv2-qRT-F	AACCTCTCGTCTTCACTCCTCTCTC
	Figla_tv2-qRT-R	TCCCTAGGCCTTCAGTCTGG
	StAR-F	AGGACGGCTGGACCACTGAAAT
	StAR-R	ACCTCGTGGGTGACCATCGTGT
	P450scc-F	GGATACGGGCGTGGTGAA
	P450scc-R	TGAATGGCCGGGTGCTTA
	β-actin-F	GCTGTGCTGTCCCTGTA
	β-actin-R	GAGTAGCCACGCTCTGTC
	Rpl13α-F	GTTTGCCCTCCTTGGTCG
	Rpl13α-R	TGCCTGCTTTGTCAGCTTGA
in situ hybridization primers	Figla_tv1-ISH-F	GAAGGATCCATGGTTACAATGGAGGTTC
	Figla_tv1-ISH-R	GATGATATCTGTCTACTTTACTGGGCTTC
	Figla_tv2-ISH-F	GAAGGATCCCATGCCCTGATGATTTCT
	Figla_tv2-ISH-R	GATGATATCTCTGCCACTACTCCACCT
